# Emotional face expression modulates occipital-frontal effective connectivity during memory formation in a bottom-up fashion

**DOI:** 10.3389/fnbeh.2015.00090

**Published:** 2015-04-23

**Authors:** Daiming Xiu, Maximilian J. Geiger, Peter Klaver

**Affiliations:** ^1^Division of Psychopathology and Clinical Intervention, Department of Psychology, University of ZurichZurich, Switzerland; ^2^Department of Psychiatry, Psychosomatics and Psychotherapy, University of WürzburgWürzburg, Germany; ^3^Center for MR Research and Child Research Center, University Children's Hospital ZurichZurich, Switzerland; ^4^Zurich Center for Integrative Human Physiology, University of ZurichZurich, Switzerland; ^5^Neuroscience Center Zurich, University of Zurich and ETH ZurichZurich, Switzerland

**Keywords:** Dynamic Causal Modeling, fMRI, facial affect, memory formation

## Abstract

This study investigated the role of bottom-up and top-down neural mechanisms in the processing of emotional face expression during memory formation. Functional brain imaging data was acquired during incidental learning of positive (“happy”), neutral and negative (“angry” or “fearful”) faces. Dynamic Causal Modeling (DCM) was applied on the functional magnetic resonance imaging (fMRI) data to characterize effective connectivity within a brain network involving face perception (inferior occipital gyrus and fusiform gyrus) and successful memory formation related areas (hippocampus, superior parietal lobule, amygdala, and orbitofrontal cortex). The bottom-up models assumed processing of emotional face expression along feed forward pathways to the orbitofrontal cortex. The top-down models assumed that the orbitofrontal cortex processed emotional valence and mediated connections to the hippocampus. A subsequent recognition memory test showed an effect of negative emotion on the response bias, but not on memory performance. Our DCM findings showed that the bottom-up model family of effective connectivity best explained the data across all subjects and specified that emotion affected most bottom-up connections to the orbitofrontal cortex, especially from the occipital visual cortex and superior parietal lobule. Of those pathways to the orbitofrontal cortex the connection from the inferior occipital gyrus correlated with memory performance independently of valence. We suggest that bottom-up neural mechanisms support effects of emotional face expression and memory formation in a parallel and partially overlapping fashion.

## Introduction

It is well-established that emotional stimuli can enhance learning (Hamann, 2001; Roozendaal and McGaugh, 2011). This enhancement has been attributed to initial encoding (Murty et al., 2010), memory consolidation (McGaugh, 2004), and retrieval processes (Sharot et al., 2004; Dolcos et al., 2005). In addition to neural interactions between the amygdala and the medial temporal lobe memory system playing a pivotal role in these processes (Dolcos et al., 2004b; LaBar and Cabeza, 2006; Smith et al., 2006; Ritchey et al., 2008), there is now increasing evidence for other neural regions contributing to the initial memory formation of emotional memories in a bottom-up and top-down manner (Dolcos et al., 2004a; Kensinger and Corkin, 2004; Mickley and Kensinger, 2008; Mather and Sutherland, 2011; Ritchey et al., 2011).

First of all, emotional stimuli can capture attention that facilitates participation of multiple regions during perception (Vuilleumier and Driver, 2007; Pessoa and Adolphs, 2010). Some of these pathways may initially bypass the amygdala and indirectly contribute to emotional memory (Kensinger and Corkin, 2004; Sergerie et al., 2005). For example, functional connectivity studies reported that emotional stimuli modulate neural activity along parallel forward pathways from visual regions to the frontal cortex, which suggests that emotional face expression facilitates perception in a bottom-up fashion. These studies do not support a mediating role of the amygdala in perception of emotional faces (Fairhall and Ishai, 2007; Dima et al., 2011). Secondly, the frontal cortex encompasses different regions that contribute to emotional memory (LaBar and Cabeza, 2006). For example, top-down connections from the orbitofrontal cortex, a region implicated in the representation of affective value, reward and behavioral guidance, have a pivotal role in emotion mediated learning (Rolls et al., 1994; Kumfor et al., 2013). Thus, while the amygdala plays a key role in rapid detection of facial affect through implicit processing (Hariri et al., 2003; Fitzgerald et al., 2006), the prefrontal cortex exerts semantic or elaborative processing via mechanisms of selective attention (Armony and Dolan, 2002). The orbitofrontal cortex not only modulates the connectivity between the amygdala and hippocampus during retrieval of emotional stimuli (Smith et al., 2006), but also constitutes a direct network with the hippocampus that mediates processing of positive emotional stimuli and increased feelings of familiarity (Mickley and Kensinger, 2008). Therefore, bottom-up activity to the orbitofrontal cortex and top-down elaborative processing of affective value in the orbitofrontal cortex on connections to the hippocampus might play important roles in the formation of emotional memories. It is however, unclear how multiple regions collaborate to support one of the two fashions and predict successful memory formation.

It should also be noted that the assumption of an automatic memory enhancement by emotional stimuli may be too simple (Bennion et al., 2013). Emotional stimuli can enhance both recall accuracy and subjective feelings of recollection (Phelps and Sharot, 2008). In conditions exerting low distinctiveness (and high inter-item relatedness) between old and new items, it was often observed that an elevation of the number of correctly identified old items was accompanied by an increase in the number of incorrect identifications of new or related items (i.e., false alarms/false memories), which means that emotional stimuli can change the response bias without improving memory performance (Dougal and Rotello, 2007; Brainerd et al., 2008). This emotion-induced recognition bias might reflect flexible criterion setting triggered by emotional valence that works to ensure that emotional stimuli are not missed or considered irrelevant (Windmann and Kutas, 2001). The emotion-induced recognition bias is less evident during conscious retrieval than during familiarity-based recognition operations (Ochsner, 2000; Johansson et al., 2004), suggesting that top-down processes play a role in rejecting emotion induced false memories. More so, for stimuli with positive affect the role of top-down processing in memory may be even more important as memories of positive stimuli depend more on gist and attention-related mechanisms (Talmi et al., 2007, 2008; Mickley and Kensinger, 2008; Mickley Steinmetz and Kensinger, 2009). Hence, when studying neural mechanisms of emotional memory, we need to take into account that emotionally valenced stimuli can influence both memory performance and response bias.

The present study utilized Dynamic Causal Modeling (DCM) of functional magnetic resonance imaging (fMRI) (Friston et al., 2003) in an incidental learning task of faces with positive (“happy”), neutral and negative (“angry” or “fearful”) emotional expressions. Our first aim was to evaluate whether bottom-up or top-down models best explain variations in neural activity during memory formation of emotional faces. Effective neural networks were characterized to elucidate the effect of emotional face expression on memory formation. In bottom-up models we hypothesized that faces with emotional expressions would engage neural pathways in a bottom-up manner to the frontal cortex (Kensinger and Corkin, 2004; Talmi et al., 2008; Dima et al., 2011). In top-down models the frontal cortex would receive stimuli with positive and negative expressions and then modulate connections to the hippocampus (Sergerie et al., 2005; Smith et al., 2006; Mickley Steinmetz and Kensinger, 2009; Ritchey et al., 2011). The best fitting model across subjects was selected and connectivity strengths were utilized to predict memory performance and response bias. Since bottom-up processes are important in perception of emotional faces (Fairhall and Ishai, 2007; Dima et al., 2011) and episodic memory formation (Dickerson et al., 2007; Sepulcre et al., 2008), we expect that the bottom-up model best explains memory formation of emotional faces. Our second aim was to examine whether pathways involved in emotional face processing directly contribute to memory performance. Based on the role of frontal and visual areas in memory formation and emotional face processing, we expect that pathways between these areas are involved in both these processes.

## Method

Eighteen healthy male adults (age 18–35 years old, mean = 27.6 years, *SD* = 5.1) without psychiatric or neurological disorders were recruited through advertisement at the university campus (University of Zurich). All subjects were German speakers, with 33.3% Swiss German speakers. They provided written informed consent and received payment for their participation. The study was in accordance with the guidelines of the local ethics review board of the Canton of Zurich.

### Experimental procedure

**Figure 1:**
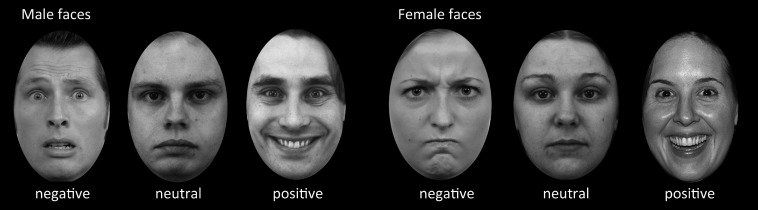
**Example stimuli used in the incidental-learning paradigm and recognition memory test**. In the learning phase, the subjects were asked to judge “how much would you like to approach this person, if you encountered this person on the road?” on a six-point scale. The example pictures are adapted from different databases such as the NimStim Face Stimulus set, Karolinska Directed Emotional Faces Database, and Radboud Faces Database.

This study investigated the influence of face expression (negative, positive, and neutral) on memory formation in an incidental-learning paradigm. Ratings on emotional valence and attractiveness were used to select the most and least attractive pictures respectively for both male and female faces with positive (“happy”), negative (“angry”) and neutral expressions (Rimmele et al., 2009; Dinkelacker et al., 2011) (examples are shown in Figure 1). The pictures of faces were an assembly from different databases: NimStim Face Stimulus set (www.macbrain.org), Karolinska Directed Emotional Faces database (KDEF; www.emotionlab.se/resources/kdef) and freely available photographers pictures (www.photo.net). These were formatted to a uniform standard grayscale pictures of adult faces with direct eye contact, cut in an ellipsoid shape on a black background. Hair, glasses, beard were allowed, but approximately equally distributed across emotional valence (Dinkelacker et al., 2011). The negative faces had angry and fearful expressions, the positive faces had happy expressions. These pictures were rated independently on a nine-point Likert scale and classified according to the valence rating (*n* = 30) in a previous study (Dinkelacker et al., 2011). The same set was also used and rated independently by Rimmele et al. (2009). This resulted in 148 faces. We added a small number of faces (20) from the Radboud Face Database with negative valence after formatting them into the same uniform standard. That database is a set of validated faces for positive, neutral and negative emotional expressions (Langner et al., 2010). Thus, the reported studies that validated these stimuli showed that on average there is a clear distinction between the valence of faces within the categories of face expression (positive, negative, and neutral). In three separate fMRI runs, subjects were presented with randomly intermixed 112 gray-scale faces of different attractiveness, valence, and gender. Each face was displayed for 3.5 s in the center of the screen. Inter-stimulus intervals varied between 2 and 18 s during which a fixation cross was shown. The tasks of the subjects was to judge “how much would you like to approach this person, if you encountered this person on the road?” and rank this judgment on a six-point scale (from “very willingly” to “very reluctantly”). For half of the subjects the buttons were ranked “1, 2, 3” for the left and “4, 5, 6” for the right hand. To minimize left/right side effects, the other half of the subjects used a reversed ranking order. Subjects were instructed to think well before deciding and to press the button when the fixation cross appeared. Subjects were not informed that this task would be followed by a memory test (Grady et al., 2002). Forty minutes after the study phase subjects completed a surprise recognition memory test outside the scanner in which 112 studies faces were intermixed with 56 new faces. For each face subjects were required to indicate by button press whether it was old or new on a six-point confidence scale (two response pads each with three buttons each ranging from “sure old” to “sure new”).

### Behavioral analysis

This study only included the behavioral reactions to old and new faces without considering confidence level. We tested if confidence predicted memory performance or response bias, but found no significant interaction between confidence level and emotion on memory performance or response bias [*F*_(4,48)_ < 1, *p* > 0.4]. This justified collapsing across confidence levels and allowed us to increase statistical power. Specifically, hit rate denoted the correct recognition proportion of studied faces for which subjects reported “sure old,” “rather old,” or “unsure old.” False alarm rate denoted the proportion of unstudied faces for which subjects incorrectly responded “sure old,” “rather old,” or “unsure old.” Hit rate and false alarm rate were calculated for each face expression separately. Faces that did not yield a response were excluded from the analysis. Memory performance [Pr = *p*(hit rate – false alarm rate)] and response bias [Br = *p*[false alarm rate/[1 – (hit rate – false alarm rate)]]] were assessed according to the two-high-threshold theory (Snodgrass and Corwin, 1988). These scores were separately calculated for faces with positive, negative and neutral expressions. Statistical analysis on behavioral data relied on a repeated measures ANOVA with face expression as factor (positive, neutral, negative). Greenhouse–Geisser corrections were applied on degrees of freedom whenever sphericity assumptions were violated. All statistical analyses were performed using SPSS 19.

### Brain imaging acquisition

Magnetic resonance imaging data were acquired on a General Electric Signa Excite 3.0 T whole-body scanner at the Center for MR Research of the University Children's Hospital Zurich. For fMRI three series of 159 scans sensitive to BOLD contrast with 44 axial slices covering the whole brain were acquired with a T2^*-sensitive^ multi-slice echo planar imaging (EPI) sequence (repetition time = 2.4 s; echo time = 32 ms; field of view = 240 cm; image matrix = 64 × 64; voxel size = 3.75 × 3.75 × 3.50 mm^3^; flip angle = 80°). The first four scans were discarded to allow for equilibration effects. Other scans were acquired that are beyond the scope of this paper.

### fMRI analysis

#### Preprocessing

Data were analyzed using SPM12b (http://www.fil.ion.ucl.ac.uk/spm/software/spm12). All volumes were slice time corrected, realigned to the first volume, corrected for motion artifacts using the ArtRepair-toolbox that detected and corrected volumes for which the signal deviated more than three standard deviations or 1 mm movement per TR (Mazaika et al., 2007), normalized into standard stereotactic space using MNI template and smoothed with a 9 mm full-width at half maximum Gaussian kernel.

#### First level analysis

For each subject, we concatenated the data from three sessions and constructed a general linear model according to the emotional valences, where vector onsets represented negative, positive, and neutral face expressions. This model was used for the DCM analysis. In addition, a separate general linear model was modeled to define volumes of interest (VOIs). This model evaluated the subsequent memory effects and was based on the recognition test. Vector onsets represented remembered faces (participants pressed “sure old,” “rather old,” or “unsure old” on old items) and forgotten faces (participants pressed “sure new,” “rather new,” or “unsure new” on old items). The subsequent memory effect was identified from the contrast “remembered faces minus forgotten faces,” and the face perception effect with all facial stimuli was identified by activity to both remembered and forgotten faces compared with baseline. Faces that yielded no responses during the recognition memory test entered the model as a regressor of no interest. All onsets of two models were convolved with a hemodynamic response function and a high-pass filter (128 s) was applied to remove low-frequency noise. Outlier parameters from the realignment procedure of the artifact-repaired data were used as covariates in the design matrix.

#### Volumes of interest

We selected priori regions of interest at the second level. Random-effects analyses of the single-subject contrast images for the subsequent memory effect model were used to identify regions related to face perception (family-wise correction *p* < 0.05) and successful memory formation (subsequent memory effect: *p* < 0.001, uncorrected) at the group level. Due to the robust effect in left hippocampus, we limited our regions of interest to the left hemisphere, which was also motivated by Smith et al. (2006). As a result, face perception related regions included the inferior occipital gyrus (IOG: *x* = −40, *y* = −78, *z* = −10) and fusiform gyrus (FUS: *x* = −36, *y* = −52, *z* = −10). A subsequent memory effect was found in several limbic and non-limbic regions (Table 1). We restricted the DCM analysis to two limbic areas [hippocampus (HPC): *x* = −30, *y* = −18, *z* = −14 and amygdala (AMG): *x* = −26, *y* = 2, *z* = −24], and two non-limbic areas related to attention and emotion processing [superior parietal lobule (SPL): *x* = −14, *y* = −68, *z* = 66 and orbital frontal cortex (OFC): *x* = 0, *y* = 62, *z* = −18]. The HPC, AMG, and OFC were expected. We included the SPL, because this region was considered to be involved in visual-spatial attention and may support both memory and emotion. For each subject, six VOIs used for the DCM analysis were defined as 4 mm spheres at the center of the nearest local maximum of group maximum, within the same anatomical area (information about centers of VOI for each subject in Supplementary Table A). The time series of each VOIs were extracted by using Eigen variates of SPM12b separately using the emotion model.

**Table 1:** **Brain regions related to successful memory formation based on the contrast between studied faces subsequently correctly recognized as old (hits) > studied faces subsequently identified as new (misses)**.

**Region (AAL)**	**Lobe**	**L/R**	**Peak coordinates**			**Cluster size**	***T*-values**	**Extend threshold (FDR)**
			***x***	***y***	***z***			
Parahippocampal g.	Limbic	L	−18	−26	−20	47	4.23	0.09^*^
Parahippocampal g./amygdala	Limbic	R	22	2	−24	87	5.34	
Amygdala (AMG)	Limbic	L	−26	2	−24	13	4.88	
Hippocampus (HPC)	Limbic	L	−30	−18	−14	12	4.31	
Posterior cingulate g.	Limbic	R	4	−44	6	153	4.81	0.025
Superior parietal lobule (SPL)/precuneus	Parietal	L	−14	−68	66	148	5.77	0.025
Orbital frontal cortex (OFC)/rectus g.	Frontal	R/L	0	62	−18	111	5.53	0.053^*^
Cerebellum 9/medulla	Cerebellum	R/L	8	−40	−52	282	6.14	0.003

Results are reported at a height threshold of p < 0.001, uncorrected. Areas outside the limbic lobe are reported only when they survived or showed a trend (

* *) after cluster extent correction (FDR p < 0.05). Regions are listed based on the largest AAL cluster according to the xjview toolbox. Abbreviations: g, gyrus*.

### Dynamic causal modeling

#### Model specification

DCM identifies dynamic and non-linear systems in the brain that capture dependencies of brain regions over time and also considers their interactions between inputs and neural activity (Friston et al., 2003). We used the emotion model in order to clarify the emotional effects on connectivities. Assuming that emotional valence mediated propagation of face processing during encoding, an initial model for all subjects included bidirectional endogenous connections between all six regions and a main effect of “all faces” as the driving input entering the visual region, IOG. According to our hypotheses, this model was differentiated into bottom-up (BU) and top-down (TD) family models (**Figure 4A**). BU family models indicate that emotion (negative and positive valences) modulated parallel forward pathways to the OFC during encoding. Emotion can influence one or more pathways from the IOG, FUS, SPL, HPC, and AMG to the OFC, which contributed to 27 bilinear models. TD family models depicted that emotion influenced the modulatory effect of the OFC on one or more connections with the hippocampus. That is, the emotional stimuli (positive and negative faces) were directly processed in the OFC. The OFC then modulated one or multiple connections from the IOG, FUS, SPL, and AMG to the HPC. The TD model family consisted of 15 non-linear models. Details about model specification are shown in Supplementary Table B. To sum up, we produced 42 variants of DCM models with 30 endogenous connections representing the functional coupling between each of the six regions. Modulatory effects consisted of five emotional effects in the bottom-up family (facial affect on connections from IOG, FUS, SPL, HPC, and AMG to OFC) and four effects of the OFC in the top-down family (the modulation from OFC on the connections from IOG, FUS, SPL, and AMG to HPC).

#### Model comparison

DCM can utilize family level inference and Bayesian model averaging (BMA) to select the model families and estimate the effective connectivities of optimal model(s) within families (Friston et al., 2003; Penny et al., 2010). Crucially, family inferences allow a large number of models to compare and provide more than one model as overwhelming winner. Family comparison and model selection was implemented using random-effects (RFX) Bayesian model selection (BMS) in SPM12b (Stephan et al., 2009; Penny et al., 2010). Two indices, the expected and exceedance probabilities, which were computed from the posterior densities over 42 models, denoted the level of confidence with which a given model outperformed any other model tested. In family inferences, the winner was selected between the BU family and TD family. Family level posteriors are a summation of model level posteriors over family members. Furthermore, in order to investigate whether the effective connectivities supported the memory formation, we applied the random effects of BMA to acquire subjects' connectivity estimates across all models based on the group winning family (Penny et al., 2010). We then used Spearman correlations to evaluate associations between behavioral measures (memory performance and response bias) and parameters for endogenous connections and modulatory effects of emotion on connections in the winning family. Since we were interested only in the connections that were relevant for emotion processing we tested only those endogenous connections and modulatory connections in the winning family that connect to the OFC in the BU model family, respectively to the HPC in the TD model family and applied Bonferroni correction accordingly.

## Results

### Behavioral results

**Figure 2:**
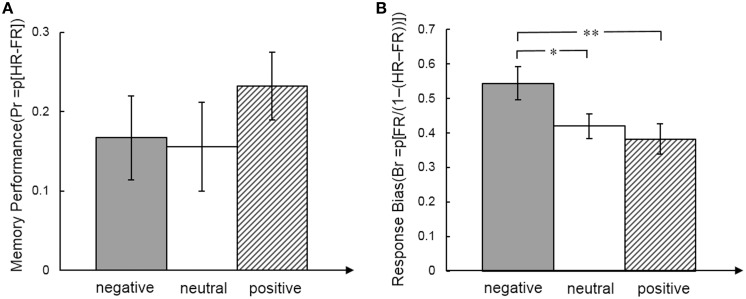
**Memory performance (A) and response bias (B) measures for emotional face expression**. *Post-hoc t*-tests indicated differences between emotional face expressions. ^*^p < .05; ^**^p < .01.

For the behavioral analysis we calculated effects of emotion on memory performance for each expression separately (Pr = HR − FR) and response bias (Br = FR/(1 − (HR-FR)) (HR, hit rate, FR, false alarm rate). Memory performance and response bias for total and for each emotional face expression separately were significantly larger than 0 (all *p* < 0.05). A repeated measures ANOVA showed no effect of face expression on memory performance [*F*_(2,34)_ = 1.05, *p* = 0.36, η^2^ = 0.058], but response bias was significantly different between emotional faces [*F*_(2, 34)_ = 6.13, *p* = 0.005, η^2^ = 0.265]. The response bias was higher for negative faces than neutral [*t*_(17)_ = 2.18, *p* = 0.044, effect size *r* = 0.323] and positive faces [*t*_(17)_ = 3.13, *p* = 0.006, effect size *r* = 0.379] (Figure 2).

### Subsequent memory effect

**Figure 3:**
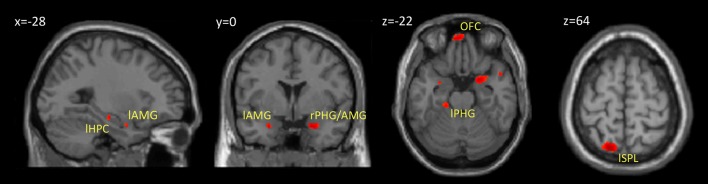
**Brain regions showing a subsequent memory effect**. The figure shows the results from contrast between subsequently recognized faces (hits) and subsequently forgotten faces (misses) (*p* < 0.001, uncorrected). Abbreviations are listed in Table 1.

Within our neuroimaging data we found a subsequent memory effect in several limbic and non-limbic areas. Limbic areas included the left hippocampus, bilateral amygdalae, left parahippocampal gyrus, and posterior cingulate gyrus. Activity outside the limbic cortex was found in the posterior cerebellum, left superior parietal lobule, and medial frontal cortex including the rectus gyrus and orbital frontal gyrus. Details are provided in Table 1 and Figure 3.

### Family comparison and model selection

**Figure 4:**
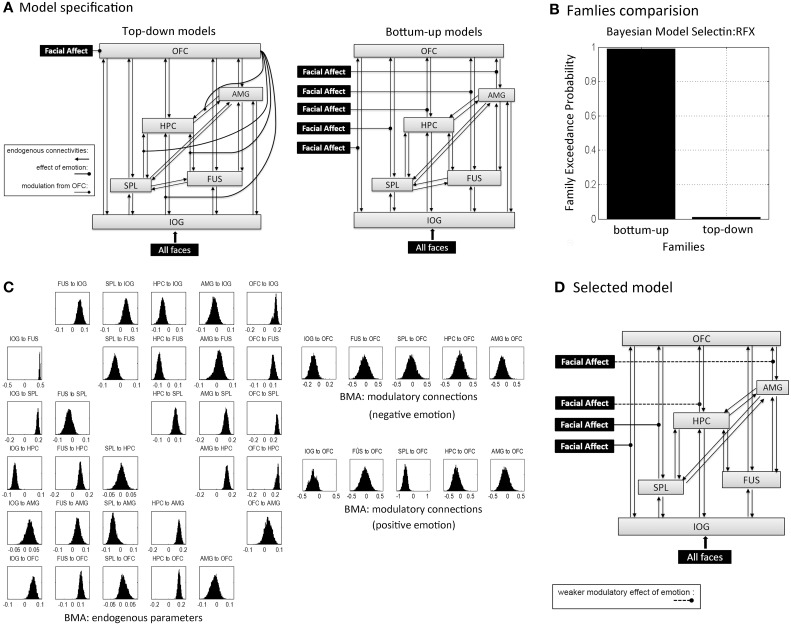
**Dynamic Causal Modeling analysis.(A)** Concept models for the effective connectivity and emotional modulations within a memory-related network during learning in terms of the top-down and bottom-up approaches. Schematically, inputs of “all faces” are in visual region IOG; the modulations of facial affects correspond to negative and positive facial expressions. Model specification was based on these two concept models with one or several modulatory pathways (see in Supplementary Table B): each one of 27 bottom-up family models had one to five bottom-up pathways that received stimuli from affective faces; all 15 top-down family models processed affective faces in OFC, but each one of top-down models received one to four modulations from OFC. **(B)** The exceedance probability for family comparison based on random effects analysis from Dynamic Causal Modeling analysis. **(C)** The posterior densities of average network parameters from random effects Bayesian model averaging for endogenous connections (left) and modulatory connections for negative and positive emotional faces respectively (right). **(D)** The selected model by DCM analysis (model 25 in Supplementary Table B); the second and fourth best-fitting models implied weaker modulatory effects on the connections from the HPC and AMG to the OFC that are showed in dashed lines.

First we executed the family comparison between bottom-up models family (totally 27 models) and top-down models family (totally 15 models). The BU family models were superior to the TD family with an exceedance probability of 99.3% across all subjects (Figure 4B). Comparing the individual 42 models, Model 25 with the highest exceedance probability of 48.1% indicated that emotion affected all pathways to the OFC except the pathway from the FUS to the OFC (Model 25 in Supplementary Table B). The second-best model, Model 24, with 19.7% exceedance probability and Model 16 with 13.8% exceedance probability indicated that the connections from AMG and HPC to OFC received weaker affective effects than IOG and SPL to OFC (Figure 4D). We then used the random effects of BMA to acquire subjects' connectivity estimates for endogenous and modulatory parameters. Figure 4C shows the posterior densities of average network parameters from random effects BMA. Of the bottom-up pathways, the IOG, FUS, and HPC had positive endogenous connections to the OFC. Within them, IOG→OFC received negative modulations from both negative and positive emotional expressions (modulatory parameter for negative expression: Median = −0.096, 25% quartile = −0.499, 75% quartile = 0.583; for positive expressions: Median = −0.095, 25% quartile = −0.578, 75% quartile = 0.259). The connection SPL→OFC received negative modulations from positive emotional expressions (Median = −0.450, 25% quartile = −1.272, 75% quartile = −0.024). The modulatory parameter estimates were negative, which indicated that an enhancement of activity associated with facial affect in the IOG and SPL resulted in suppression of activity in the OFC.

### Correlations between connectivity estimates and behavior

**Figure 5:**
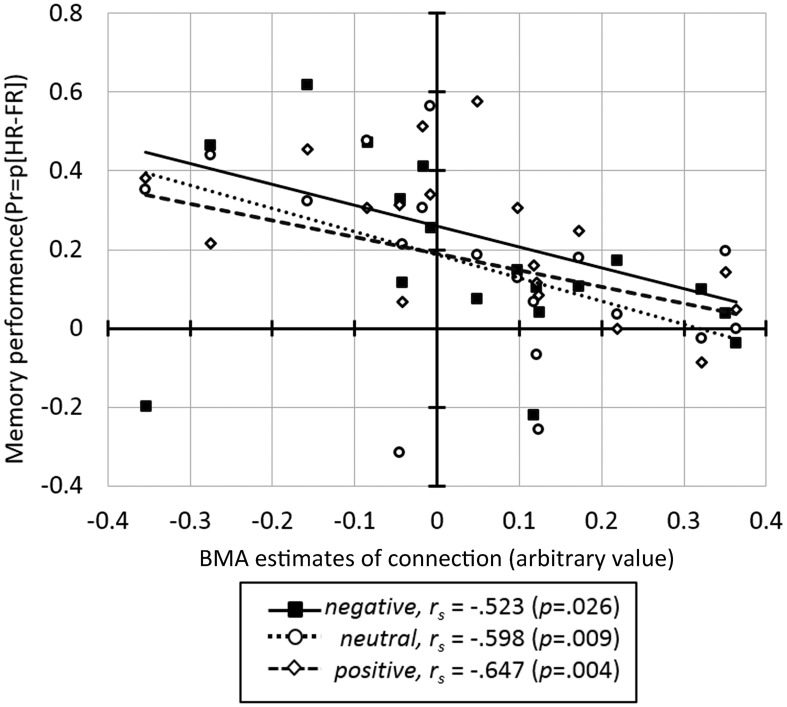
**The correlation between the BMA parameter estimates for the IOG→OFC connection and memory performance for faces in three emotional expressions**.

Correlation analysis between the BMA estimates of BU endogenous connections and behavioral measures across all subjects revealed a significant negative correlation between memory performance and the IOG→OFC pathway (*r_s_* = −0.680, *p* = 0.002). This correlation was found for faces with all emotional expressions (correlation with Pr-negative: *r_s_* = −0.523, *p* = 0.026; Pr-neutral: *r_s_* = −0.598, *p* = 0.009; Pr-positive: *r_s_* = −0.647, *p* = 0.004; Figure 5) and survived Bonferroni correction (α < 0.0033, see Table 2). This negative correlation indicated that neural activity in the IOG elicited an inhibition of activity in the OFC in high performers, whereas it yields a facilitation of activity in the OFC of low performers. We found no significant correlation between bottom-up endogenous parameters and response bias. As for the modulatory parameters, the effects of negative stimuli occurred in the correlations between connection SPL→OFC with response bias for negative faces (*r_s_* = 0.482, *p* = 0.043) and connection AMG→OFC with total response bias (*r_s_* = 0.482, *p* = 0.043). These correlations, however, did not survive Bonferroni correction.

**Table 2:** **Median and quartiles DCM endogenous parameters and modulatory estimates based on Bayesian model averaging (BMA) across all subjects and all models, and Spearman rho correlation (*r_s_*) between parameters and behavioral performances**.

	**Median**	**Quartiles**		**Memory performance (*r_s_*)**				**Response bias (*r_s_*)**			
		**25%**	**75%**	**Total**	**Negative**	**Neutral**	**Positive**	**Total**	**Negative**	**Neutral**	**Positive**
**ENDOGENOUS PARAMETERS**											
IOG→OFC	0.073	−0.055	0.184	−0.680^**^	−0.523^*^	−0.598^**^	−0.647^**^	0.026	0.228	−0.358	−0.098
FUS→OFC	0.047	−0.065	0.268	−0.067	0.018	−0.038	−0.131	0.280	−0.094	0.044	−0.333
SPL→OFC	0.047	−0.103	0.134	0.013	0.090	−0.079	0.036	0.034	−0.092	0.028	0.401
HPC→OFC	0.247	0.038	0.317	−0.201	−0.026	−0.174	−0.199	−0.255	−0.265	−0.011	0.015
AMG→OFC	−0.085	−0.145	0.175	−0.156	−0.119	0.038	−0.311	0.067	0.302	−0.230	0.234
**MODULATORY ESTIMATES**											
IOG→OFC, negative	−0.096	−0.499	0.583	0.321	−0.106	0.459	0.321	0.267	0.253	−0.040	−0.077
FUS→OFC, negative	−0.054	−0.342	0.109	0.267	0.232	0.046	0.451	−0.098	−0.373	−0.218	0.104
SPL→OFC, negative	0.038	−1.216	0.598	−0.207	−0.181	−0.042	−0.224	0.141	0.482^*^	0.300	0.094
HPC→OFC, negative	0.011	−0.277	0.534	0.011	0.121	−0.162	−0.003	−0.040	−0.317	−0.170	−0.129
AMG→OFC, negative	−0.442	−0.709	0.058	−0.152	−0.298	−0.133	−0.069	0.482^*^	0.284	0.162	0.280
IOG→OFC, positive	−0.095	−0.578	0.259	0.034	0.082	0.273	−0.358	−0.331	0.172	−0.422	−0.046
FUS→OFC, positive	−0.008	−0.270	0.266	0.222	0.038	0.311	0.015	−0.240	−0.290	−0.385	−0.331
SPL→OFC, positive	−0.450	−1.272	−0.024	0.022	0.112	0.119	−0.282	−0.185	0.187	−0.187	−0.437
HPC→OFC, positive	−0.027	−0.823	0.245	0.154	−0.059	0.131	0.278	0.185	−0.185	0.071	0.123
AMG→OFC, positive	−0.101	−0.564	0.166	−0.232	−0.063	−0.249	−0.166	0.309	0.247	0.007	−0.005

* p < 0.05;

** *p < 0.0033, Bonferroni correction for multiple comparison (5 endogenous parameters and 10 modulatory parameters)*.

## Discussion

This study aimed at examining how emotional face expression is implemented in neural networks supporting memory formation of faces. We utilized DCM of fMRI to study effective connectivities during face encoding and compared “bottom-up” and “top-down” models that describe the influence of emotion on memory formation. In accordance with the theory that emotion operates during memory formation via multiple regions participating in perceptual, attentional, or semantic processes (LaBar and Cabeza, 2006), our DCM analysis was implemented in an extended network combining facial perception and memory formation related areas. Specifically, subsequently remembered faces were associated with higher activations compared to subsequently forgotten faces not only in limbic areas conveying the hippocampus, amygdala, and posterior cingulate gyrus, but also in the superior parietal lobe, orbitofrontal cortex and cerebellum. Whereas, limbic and orbitofrontal areas are frequently reported in the context of emotional memory operations, the superior parietal lobe and cerebellum are less often discussed. The superior parietal lobule is a region that can provide (spatial) attentional assistance during perception and memory processing (Hoffman and Haxby, 2000; Ciaramelli et al., 2008; Hutchinson et al., 2009; Uncapher and Rugg, 2009; von Allmen et al., 2013). The posterior cerebellum has been also recognized in prospective cognitive and affective processing beyond strict motor planning (Schmahmann and Sherman, 1998; Cotterill, 2001; Chen et al., 2014).

The “bottom-up” model, in which emotion exerted effects along multiple parallel feed-forward pathways to the orbitofrontal cortex, prevailed across all subjects. This finding is in line with our hypothesis and suggests that emotion exerts parallel effects on multiple forward pathways to the prefrontal cortex (i.e., IOG→OFC, SPL→OFC, HPC→OFC, and AMG→OFC). The OFC has been associated with elaborative processing of valence and reward (O'Doherty et al., 2001; Kringelbach, 2005), which was tested in our top-down family models. However, the winning family of bottom-up models corresponds to the view that emotional stimuli are processed simultaneously along “many roads” across the face-processing network (Kensinger and Corkin, 2004; Pessoa and Adolphs, 2010). Furthermore, the results of model selection highlighted the effective connectivities from IOG and SPL to OFC, as these connections were present in all preferred models. Previous studies showed that the inferior fronto-occipital fascicle and superior longitudinal fascicle connect the visual system with the frontal cortex along dorsal and ventral pathways (Johnson et al., 1996; Martino et al., 2010; Sarubbo et al., 2013). The inferior connections build the ventral visual stream and engage functional coupling between visual and inferior prefrontal cortices supporting visual attention and perception (Gregoriou et al., 2009), while the superior connections extend upon the dorsal visual stream and connects to dorsal parts of the prefrontal cortex. The superior parietal lobe does not seem to have direct connections to the orbitofrontal cortex, but can provide attentional assistance for face perception during gaze perception (Hoffman and Haxby, 2000), memory encoding (Uncapher and Rugg, 2005), retrieval (Ciaramelli et al., 2008), and working memory in alignment with hippocampal activity (Ranganath and D'Esposito, 2001; Nee and Jonides, 2013; von Allmen et al., 2013) and frontal regions (Olesen et al., 2003). The modulatory effect of emotion on the IOG-OFC and SPL-OFC connectivities in our task, might suggest that emotion modulates visual processes along the dorsal and ventral visual system during memory formation. The posterior densities of the modulatory BMA parameters tended to be negative, which would indicate that activity induced by emotionally valenced stimuli in the IOG resulted in suppression of activity in the OFC. Since modulatory BMA parameters were negative for positively and negatively valenced faces, it is possible that the OFC is actively suppressed by connecting regions as soon as emotional information is presented. This active suppression might prevent that emotional information does not distract from processing the facial features during the evaluation of approachability in the incidental learning task. It should be noted here, that modulation of the pathways to the OFC are independent from the intrinsic connections with the OFC.

Our second aim was to evaluate whether connectivities predict successful memory formation for emotionally valenced faces. Of all pathways to the OFC within the BU model we found that only the IOG to OFC endogenous connection negatively correlated with memory performance. All three expressions showed a similar correlation with endogenous connectivity. This means that for subjects with higher performance neural activity in the IOG caused a suppression of neural activity in the OFC, whereas in low performers activity in the IOG caused a facilitation of activity in the OFC. This mechanism was slightly more pronounced for positive face expressions, but there was no difference in correlation coefficients for the different emotional expressions. On average, there was a weak positive connectivity from the IOG to the OFC, as illustrated in Figure 4C, yet our results suggest that individual differences on the signal transfer between the IOG and OFC is associated with subsequent memory performance. One potential explanation for this effect might be that a higher decoupling between the visual processing areas and the frontal cortex is supportive during memory formation, because it may prevent the frontal cortex from being overloaded during visual processing. Several studies reported increased functional coupling in resting state functional connectivity MRI between the hippocampus and the frontal cortex during and immediately after learning (Ranganath et al., 2005; Tambini et al., 2010), and between visual areas and the frontal cortex immediately after learning (Stevens et al., 2010). Few studies effectively investigated neural coupling during episodic memory formation, except for a few intracranial EEG studies that show coupling and decoupling between brain regions relevant for memory formation (Fell et al., 2001; Axmacher et al., 2008; Sehatpour et al., 2008). These studies suggested that sustained neural decoupling follows transient coupling between visual and hippocampal regions during successful memory formation. As far as we know such mechanisms have not yet been demonstrated between occipital and frontal regions. Yet, one might speculate that decoupling can follow transfer of information during coupling of neural networks. Such a mechanism might prevent that sensory information interferes with higher order processing of information. Thus, although evidence is still sparse, we tentatively suggest that the likelihood for memory formation to occur increases when the orbitofrontal cortex is temporary decoupled during evaluation of faces with different emotional expressions. Another point to note is that we found no differential effect of emotional valence on the association between connectivity and memory formation. It is important to remind, however, that we found no differential effect of emotional valence on memory performance either, so that inferences between emotional memory and connectivity cannot be drawn without further investigation. Taken together, model selection indicated that emotion modulated the IOG to OFC connection, while individual differences in memory performance were associated with endogenous connection strength, independently of emotional face expression. We thus suggest that the connectivity results tend to be in line with other views that emotion exerts parallel effects on perception (Calder and Young, 2005), attention and memory (Talmi et al., 2008, 2013).

DCM of fMRI provided an effective approach to investigate the effects of emotional face expression on memory formation. It should, however, be noted that combining and comparing Bayesian statistics with classical statistical approaches underlies limitations for interpretation of the data because DCM uses full time courses to estimate best fitting models, whereas correlations with connectivity estimates has much less statistical power. Nevertheless, we had a conservative classical statistical approach and sufficient statistical power to infer that connectivity estimates for the IOG→OFC were reliably related to memory performance. We also found that emotional face expression affected response bias, but not memory performance, which is in line with numerous recognition memory studies for emotional stimuli (Windmann and Kutas, 2001; Johansson et al., 2004; Dougal and Rotello, 2007; Brainerd et al., 2008). There is now some evidence that recognition memory operations might account for emotion induced differences in response bias at the behavioral and neural level. For example, emotion-induced recognition bias was associated with differences in frontal ERPs during recognition memory (Johansson et al., 2004). Similar to our study the authors reported no effect emotion induced enhancement of memory performance and only reported emotion induced enhancement of response bias at the behavioral level. Negative pictures also enhanced recollective experience, but not contextual detail of memory, and this recollective experience related to amygdala activity (Sharot et al., 2004). Enhanced focusing to specific details during recollection was also reported to induce recollective experience (Sharot et al., 2008). When negative faces induce enhanced focusing to a salient visual feature (i.e., the negative expression), independently of whether these items were new or old, this might elicit the phenomenon of recollective experience, even when a negative face is new. So all this evidence suggests that emotion induced response bias for negative faces relates to recognition operations rather than to memory formation processes that were studied here.

It should also be noted that the sample included healthy young male adults. Some studies included females and found that menstrual cycle influenced neural responses on emotional stimuli (Protopopescu et al., 2005). We chose to measure males to induce lower variance in the behavioral and neural data and to avoid variability in the data by factors we could not control for. It needs to be resolved whether our main results can be replicated in different samples, such as females, different age groups, or clinical samples for which similar networks were implicated (e.g., stress disorders, prosopagnosia) (Brewin et al., 2010; Dinkelacker et al., 2011). Moreover, our study investigated whether emotion contributed to the effective connectivity based on a network encompassing positive subsequent memory effects (remembered > forgotten). Whether emotion affects effective connectivities related to areas involved in forgetting (Daselaar et al., 2004) is another interesting topic that can be further investigated in future studies. Finally, our stimulus set included pictures of faces that had moderate valence. It is currently unclear if our main results hold under conditions of higher arousal induced by the stimuli, or by circumstantial information such as in real-world situations. Although several studies suggested that higher arousal captures attention and engages top-down elaborative processes (Dolcos et al., 2004a; Ritchey et al., 2011), it remains to be investigated whether such situations would also induce more top-down processing relative to bottom-up processing in effective connectivity. Taken together, we are confident to suggest that the pathways involved in modulating memory networks by emotion and pathways that successfully contribute to memory formation of emotional faces are partially overlapping and work in parallel in a bottom-up fashion.

## Author contributions

PK and MG conceptualized and designed the study; MG acquired the data; DX, MG, and PK analyzed the data; DX, PK wrote the paper.

### Conflict of interest statement

The authors declare that the research was conducted in the absence of any commercial or financial relationships that could be construed as a potential conflict of interest.
