# Supplementary material for: Emotional face expression modulates occipital-frontal effective connectivity during memory formation in a bottom-up fashion
Source: Front Behav Neurosci. 2015 Apr 23;9:90. doi: 10.3389/fnbeh.2015.00090 (PMC4407577; doi:10.3389/fnbeh.2015.00090)
Supplement: Supplementary file 1 [file Table1.DOCX]

**Supplement Table A**. MNI coordinate of Volumes of interest for each subjects

|  | IOG | | | | FUS | | | | SPL | | | |
| --- | --- | --- | --- | --- | --- | --- | --- | --- | --- | --- | --- | --- |
|  | Peak coordinate | | | d  (mm) | Peak coordinate | | | d  (mm) | Peak coordinate | | | d  (mm) |
|  | x | y | z |  | x | y | z |  | x | y | z |  |
| Group maximum | -40 | -78 | -10 |  | -36 | -52 | -10 |  | -14 | -68 | 66 |  |
| subject 01 | -36 | -80 | -18 | 9.17 | -40 | -56 | -22 | 13.27 | -8 | -66 | 62 | 7.48 |
| subject 02 | -32 | -82 | -14 | 9.80 | -38 | -46 | -18 | 10.20 | -10 | -66 | 62 | 6.00 |
| subject 03 | -36 | -68 | -14 | 11.49 | -38 | -62 | -16 | 11.83 | -12 | -69.27 | 65.27 | 2.48 |
| subject 04 | -28 | -80 | -12 | 12.33 | -34 | -60 | -16 | 10.20 | -13.94 | -67.81 | 65.94 | 0.21 |
| subject 05 | -34 | -68 | -16 | 13.11 | -44 | -48 | -24.13 | 16.72 | -8 | -64 | 68 | 7.48 |
| subject 06 | -36 | -78 | -16 | 7.21 | -36 | -50 | -20 | 10.20 | -11.92 | -69.62 | 65.46 | 2.69 |
| subject 07 | -38 | -86 | -8 | 8.49 | -44 | -44 | -16 | 12.81 | -15.85 | -67.54 | 65.54 | 1.96 |
| subject 08 | -38 | -84 | -16 | 8.72 | -42 | -48 | -20 | 12.33 | -25.94 | -67.94 | 59.94 | 13.39 |
| subject 09 | -42 | -82 | -6 | 6.00 | -46.23 | -55.62 | -3.54 | 12.63 | -21.44 | -65.11 | 65.11 | 8.03 |
| subject 10 | -44 | -78 | 4 | 14.56 | -46 | -60 | -20 | 16.25 | -14 | -64 | 66 | 4.00 |
| subject 11 | -22 | -88 | -10 | 20.59 | -36.06 | -52.06 | -16.06 | 6.06 | -17.91 | -71.30 | 61.39 | 6.89 |
| subject 12 | -32 | -72 | -12 | 10.20 | -40 | -46 | -20 | 12.33 | -8 | -66 | 66 | 6.32 |
| subject 13 | -42 | -78 | -12 | 2.83 | -38 | -50 | -22 | 12.33 | -17.44 | -71.11 | 61.11 | 6.74 |
| subject 14 | -40 | -84 | -12 | 6.32 | -38 | -50 | -22 | 12.33 | -12 | -63.64 | 69.64 | 6.02 |
| subject 15 | -36 | -74 | -14 | 6.93 | -36 | -50 | -20 | 10.20 | -14 | -67.64 | 65.64 | 0.51 |
| subject 16 | -42 | -78 | -10 | 2.00 | -34 | -64 | -14 | 12.81 | -15.93 | -63.63 | 67.56 | 5.02 |
| subject 17 | -42 | -80 | -12 | 3.46 | -38 | -62 | -18 | 12.96 | -15.7 | -65.3 | 67.1 | 3.37 |
| subject 18 | -36 | -76 | -12 | 4.90 | -34.09 | -42.64 | -17.64 | 12.23 | -12 | -66 | 62 | 4.90 |

|  | HPC | | | | AMG | | | | OFC | | | |
| --- | --- | --- | --- | --- | --- | --- | --- | --- | --- | --- | --- | --- |
|  | Peak coordinate | | | d  (mm) | Peak coordinate | | | d  (mm) | Peak coordinate | | | d  (mm) |
|  | x | y | z |  | x | y | z |  | x | y | z |  |
| Group maximum | -30 | -18 | -14 |  | -26 | 2 | -24 |  | 0 | 62 | -18 |  |
| subject 01 | -34 | -18 | -16 | 4.47 | -28 | 4 | -24 | 2.83 | -4 | 62.84 | -19.47 | 4.35 |
| subject 02 | -28 | -18 | -12 | 2.83 | -28 | -2 | -20 | 6.00 | -8 | 60 | -18 | 8.25 |
| subject 03 | -32 | -16 | -14 | 2.83 | -26 | -2 | -22 | 4.47 | 4 | 58 | -22 | 6.93 |
| subject 04 | -32 | -18 | -12 | 2.83 | -22.38 | 3.43 | -25.14 | 4.06 | -2.4 | 65 | -17.5 | 3.87 |
| subject 05 | -32 | -20 | -16 | 3.46 | -28 | 0 | -26 | 3.46 | 6 | 58 | -24 | 9.38 |
| subject 06 | -32 | -18 | -12 | 2.83 | -24 | 4 | -22 | 3.46 | 0 | 67.04 | -14 | 6.44 |
| subject 07 | -32 | -18 | -18 | 4.47 | -28 | -2 | -22 | 4.90 | 2 | 60 | -16 | 3.46 |
| subject 08 | -26 | -20 | -12 | 4.90 | -28 | 2 | -20 | 4.47 | -4 | 64.84 | -17.47 | 4.94 |
| subject 09 | -32 | -20 | -16 | 3.46 | -22.29 | 3.86 | -25.64 | 4.47 | 0 | 60 | -16 | 2.83 |
| subject 10 | -30 | -20 | -14 | 2.00 | -22.13 | 1.93 | -23.73 | 3.88 | -2.1 | 63 | -15.4 | 3.49 |
| subject 11 | -26 | -18 | -16 | 4.47 | -28 | 0 | -26 | 3.46 | 7.91 | 63.30 | -17.39 | 8.04 |
| subject 12 | -28 | -20 | -14 | 2.83 | -24 | 0 | -26 | 3.46 | 4.11 | 66.78 | -15.44 | 6.80 |
| subject 13 | -24 | -24 | -18 | 9.38 | -26 | 2 | -24 | 0.00 | -6 | 60 | -18 | 6.32 |
| subject 14 | -30 | -20 | -12 | 2.83 | -24 | 2 | -26 | 2.83 | 8 | 61.82 | -21 | 8.55 |
| subject 15 | -30 | -16 | -14 | 2.00 | -22 | 6 | -24 | 5.66 | 6 | 62 | -18 | 6.00 |
| subject 16 | -34 | -18 | -16 | 4.47 | -24 | 0 | -26 | 3.46 | 0 | 65.36 | -15.6 | 4.13 |
| subject 17 | -34 | -18 | -14 | 4.00 | -28 | -2 | -20 | 6.00 | 2.08 | 61.50 | -21.33 | 3.96 |
| subject 18 | -22 | -30 | -26 | 18.76 | -28 | 4 | -22 | 3.46 | -2 | 60 | -16 | 3.46 |

Note: d is the distance from the individual peak coordinate to the group peak maximum
