# Supplementary material for: Emotional face expression modulates occipital-frontal effective connectivity during memory formation in a bottom-up fashion
Source: Front Behav Neurosci. 2015 Apr 23;9:90. doi: 10.3389/fnbeh.2015.00090 (PMC4407577; doi:10.3389/fnbeh.2015.00090)
Supplement: Supplementary file 2 [file Table2.DOCX]

**Supplement Table B.** Model Specification and Comparison

| Model specification | | | | | | Model Comparison based on RFX (*p*) | |
| --- | --- | --- | --- | --- | --- | --- | --- |
| Bottom-up models family | Emotional Input On Bottom-up Pathways | | | | |  |  |
|  | IOG→ OFC | FUS→OFC | SPL→OFC | HPC→OFC | AMG→OFC | Expected probablity | Exceedance probablity |
| Model 1 | ✓ |  |  |  |  | .002 | 0 |
| Model 2 |  | ✓ |  |  |  | .004 | <.001 |
| Model 3 |  |  | ✓ |  |  | .003 | 0 |
| Model 4 |  |  |  | ✓ |  | .002 | 0 |
| Model 5 |  |  |  |  | ✓ | .002 | 0 |
| Model 6 | ✓ | ✓ |  |  |  | .006 | .001 |
| Model 7 | ✓ |  | ✓ |  |  | .009 | .003 |
| Model 8 | ✓ |  |  | ✓ |  | .003 | 0 |
| Model 9 | ✓ |  |  |  | ✓ | .004 | .001 |
| Model 10 |  | ✓ | ✓ |  |  | .002 | 0 |
| Model 11 |  | ✓ |  | ✓ |  | .003 | <.001 |
| Model 12 |  | ✓ |  |  | ✓ | .003 | <.001 |
| Model 13 |  |  | ✓ | ✓ |  | .003 | <.001 |
| Model 14 |  |  | ✓ |  | ✓ | .003 | <.001 |
| Model 15 |  |  |  | ✓ | ✓ | .003 | 0 |
| Model 16 | ✓ | ✓ | ✓ |  |  | .104 | .138 |
| Model 17 | ✓ | ✓ |  | ✓ |  | .005 | .001 |
| Model 18 | ✓ | ✓ |  |  | ✓ | .005 | .001 |
| Model 19 | ✓ |  | ✓ | ✓ |  | .013 | .005 |
| Model 20 | ✓ |  |  | ✓ | ✓ | .004 | .000 |
| Model 21 |  | ✓ | ✓ | ✓ |  | .003 | .000 |
| Model 22 |  | ✓ | ✓ |  | ✓ | .006 | .001 |
| Model 23 |  |  | ✓ | ✓ | ✓ | .003 | .000 |
| Model 24 | ✓ | ✓ | ✓ | ✓ |  | .137 | .197 |
| Model 25 | ✓ |  | ✓ | ✓ | ✓ | .345 | .481 |
| Model 26 |  | ✓ | ✓ | ✓ | ✓ | .006 | .000 |
| Model 27 | ✓ | ✓ | ✓ | ✓ | ✓ | .111 | .159 |
| Top-down models family | Inputs on OFC | Top-Down Modulation from the OFC | | | |  |  |
|  |  | IOG→HPC | FUS→HPC | SPL→HPC | AMG→HPC |  |  |
| Model 28 | ✓ |  |  |  |  | .004 | 0 |
| Model 29 | ✓ | ✓ |  |  |  | .009 | .001 |
| Model 30 | ✓ |  | ✓ |  |  | .013 | .001 |
| Model 31 | ✓ |  |  | ✓ |  | .007 | <.001 |
| Model 32 | ✓ |  |  |  | ✓ | .007 | 0 |
| Model 33 | ✓ | ✓ | ✓ |  |  | .016 | .001 |
| Model 34 | ✓ | ✓ |  | ✓ |  | .015 | .001 |
| Model 35 | ✓ | ✓ |  |  | ✓ | .016 | .001 |
| Model 36 | ✓ |  | ✓ | ✓ |  | .014 | .001 |
| Model 37 | ✓ |  | ✓ |  | ✓ | .013 | .001 |
| Model 38 | ✓ |  |  | ✓ | ✓ | .014 | .001 |
| Model 39 | ✓ | ✓ | ✓ | ✓ |  | .019 | .001 |
| Model 40 | ✓ | ✓ | ✓ |  | ✓ | .021 | .002 |
| Model 41 | ✓ |  | ✓ | ✓ | ✓ | .020 | .001 |
| Model 42 | ✓ | ✓ | ✓ | ✓ | ✓ | .016 | .002 |
